# Characteristics of Thoron (^220^Rn) and Its Progeny in the Indoor Environment

**DOI:** 10.3390/ijerph17238769

**Published:** 2020-11-25

**Authors:** Shinji Tokonami

**Affiliations:** Institute of Radiation Emergency Medicine, Hirosaki University, Hirosaki 036-8564, Aomori, Japan; tokonami@hirosaki-u.ac.jp; Tel.: +81-172-39-5404

**Keywords:** thoron, thoron progeny, indoor environment, measurement technique, radioactivity, dose assessment

## Abstract

The present paper outlines characteristics of thoron and its progeny in the indoor environment. Since the half-life of thoron (^220^Rn) is very short (55.6 s), its behavior is quite different from the isotope radon (^222^Rn, half-life 3.8 days) in the environment. Analyses of radon and lung cancer risk have revealed a clearly positive relationship in epidemiological studies among miners and residents. However, there is no epidemiological evidence for thoron exposure causing lung cancer risk. In contrast to this, a dosimetric approach has been approved in the International Commission on Radiological Protection (ICRP) Publication 137, from which new dose conversion factors for radon and thoron progenies can be obtained. They are given as 16.8 and 107 nSv (Bq m^−3^ h)^−1^, respectively. It implies that even a small quantity of thoron progeny will induce higher radiation exposure compared to radon. Thus, an interest in thoron exposure is increasing among the relevant scientific communities. As measurement technologies for thoron and its progeny have been developed, they are now readily available. This paper reviews measurement technologies, activity levels, dosimetry and resulting doses. Although thoron has been underestimated in the past, recent findings have revealed that reassessment of risks due to radon exposure may need to take the presence of thoron and its progeny into account.

## 1. Introduction

Radon (^222^Rn), thoron (^220^Rn) and their progeny can be regarded as the largest contributor annually to an effective dose for the public globally [1,2]. According to the United Nations Scientific Committee on the Effects of Atomic Radiation (UNSCEAR) 2008 report, an annual effective dose from natural radiation sources is calculated to be 2.4 mSv as the worldwide average, whereas radon and thoron contribute 1.2 and 0.1 mSv, respectively. When they are inhaled, although radon and thoron gases are not significant, their progeny particularly affect the lung tissue due to alpha particles emitted in their decay chains deposited in the airways. In the past, lung cancer incidence had been found only among miners as shown in many epidemiological studies, whereas recent investigations have revealed that even indoor radon resulted in lung cancer among residents [3]. These surveys were carried out in Europe, North America and China. Therefore, the World Health Organization (WHO) issued a handbook where special attention was paid to indoor radon [3]. Subsequently, the International Commission on Radiological Protection (ICRP) has recently published two publications and one statement related to radon. In these documents, the upper value of the reference level for radon gas in homes was revised downward from the value in the 2007 Recommendations of 600 Bq m^−3^ to 300 Bq m^−3^ [4,5]. The International Atomic Energy Agency (IAEA) revised the previous Basic Safety Standard (BSS) in the same manner as the ICRP and a related guide was issued [6,7]. The WHO further advised a reference level of 100 Bq m^−3^ though it may be impossible to achieve such a low radon gas concentration in many countries. Such recommendations depend on results of an indoor radon survey. In most cases, these surveys were carried out using passive radon monitors so as to obtain an annual indoor radon concentration. Even in epidemiological surveys, the same type of radon monitor was used, because lung cancer incidence was closely related to long-term exposure to radon. Previous recommendations were given based on not the dosimetric, but on the epidemiological approach. It had been previously believed that the epidemiological approach was more reliable than the dosimetric. In ICRP Publication 65, the risk estimate was given based on the epidemiological approach [8]. As was concluded according to studies of miners, however, the conversion convention, though scientifically vague, needed to be used when applied to indoor radon studies. There was a large difference between the two approaches by a factor of more than three and many technical issues to be solved. After the data analyses on the indoor radon and lung cancer study were vigorously carried out, the risk estimates in residential radon studies were eventually concluded without using the conversion convention and came close to those given by the dosimetric approach. This is why many authoritative publications were issued and revised. However, they still state that the effect of thoron is negligible compared to that of radon, though the amount of related data is limited. It should be noted that measurement techniques for thoron are not so easy as those for radon. As the half-life of thoron atoms is much shorter than that of radon, they immediately decay, followed by ^216^Po with a half-life much shorter than thoron. A question arises here. Many passive radon monitors have been used in both national and epidemiological surveys. If thoron is present together with radon, are these well designed so as to effectively detect radon only? If high diffusion barriers are used, they depress the detection of thoron. Otherwise they may mislead and lead to wrong calculation of radon concentrations. In epidemiological surveys, this will result in incorrect lung cancer risk estimates. Most passive radon monitors have never been examined from the viewpoint of thoron interference on radon measurements. Limited data on thoron is given in UNSCEAR reports and indoor thoron surveys have never been systematically conducted. It is well known that there is no epidemiological evidence for thoron risk related to lung cancer. The risk can be estimated based only on the dosimetric approach. Under the current situation in which the dosimetric approach has become more reliable, it is important to know how large the total lung cancer risk is when influenced by thoron and its progeny. This paper comprehensively describes characteristics of thoron and its progeny in the indoor environment from the viewpoint of measurements, dose assessment and health risk.

## 2. Physical Property and Behavior

**Figure 1:**
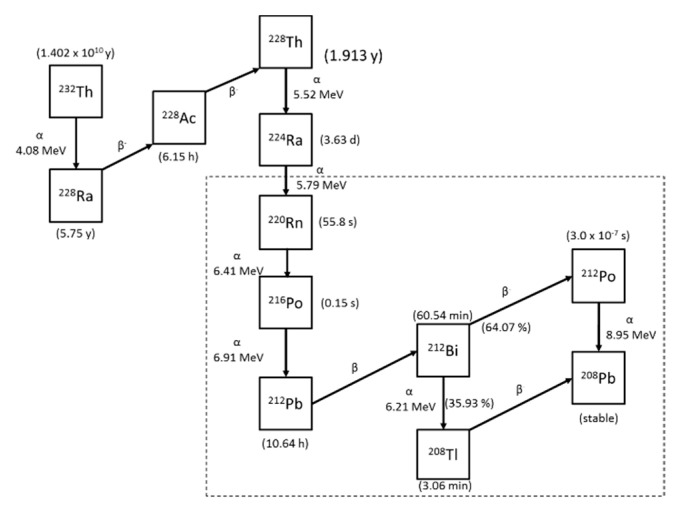
Radioactive decay series for thorium-232.

Figure 1 illustrates the radioactive decay series for thorium-232 [9] where the half-life and emitted energies are given. After Ra-224 decays, Rn-220 is formed. It is commonly called thoron, an inert gas. In the uranium-238 decay series, on the other hand, radon-222 is formed as an inert gas. There is a great deal of difference in the half-life between radon-220 and radon-222. Although Po-216 is formed with alpha decay of Rn-220, it can almost be regarded as a gas because its half-life is very short. Subsequently Pb-212 and Bi-212 are formed, which need mainly to be considered for dose assessment when they are inhaled. These concentrations are collectively expressed in the equilibrium equivalent concentration (EEC). The EEC for thoron progeny (Equilibrium Equivalent Thoron Concentration: EETC [Bq m^−3^]) can be approximately calculated by the Equation (1) after considering the contribution of Po-216:

(1) $$\mathrm{EETC}=0.913\times C_{B}+0.087\times C_{C}$$

where *C*_B_: Pb-212 is activity concentration [Bq m^−3^]; *C*_C_: Bi-212 is activity concentration [Bq m^−3^]. If the equilibrium factor for thoron (*F*_Tn_) is defined in the same manner for radon, it can be expressed as the Equation (2):

(2) $$wF_{\mathrm{Tn}}\;=\;\frac{\mathrm{EETC}}{C_{\mathrm{Tn}}}$$

where *C*_Tn_ is thoron concentration [Bq m^−3^]. The significance of the equilibrium factor for thoron is discussed in this paper from the viewpoint of dose assessment.

**Figure 2:**
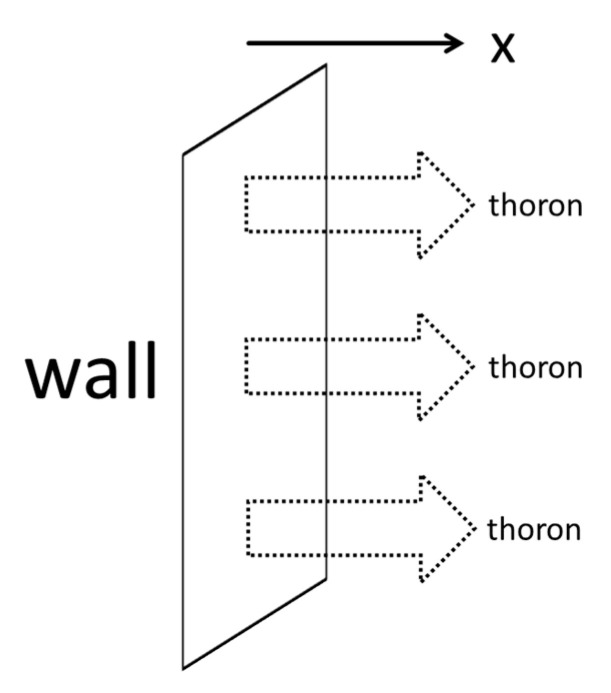
Exhalation process of thoron from macro surface.

Figure 2 exemplifies the exhalation process of thoron from macro surfaces such as walls containing its parent nuclide ^224^Ra. The exhalation and diffusion of thoron is approximately described as a one-dimensional phenomenon. When the exhalation rate of thoron from the wall is considered, for instance, the indoor thoron concentration (*C*_Tn_(*x*) [Bq m^−3^]) at distance *x* from the wall can be expressed by the Equation (3) [10,11]:

(3) $$C_{\mathrm{Tn}}\left(x\right)=\frac{E_{\mathrm{Tn}}}{\sqrt{\lambda_{\mathrm{Tn}}D}}e^{\left(-\sqrt{\lambda_{\mathrm{Tn}}/D}x\right)}$$

where *E*_Tn_ is surface exhalation rate of thoron from the wall [Bq m^−2^ s^−1^]; *λ*_Tn_ is decay constant of thoron [s^−1^]; and *D* is diffusion coefficient of thoron [m^2^ s^−1^]. If the thoron concentrations are measured at two different locations, respectively, the exhalation rate of thoron can be estimated. As the half-life of Po-216 is much shorter than that of the parent nuclide thoron, there is a radioactive equilibrium between the two isotopes.

**Figure 3:**
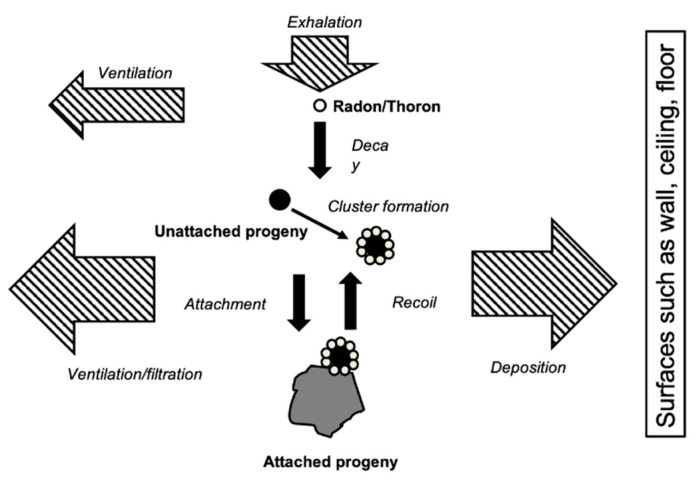
Behavior of radon/thoron and their progeny in indoor air.

**Figure 4:**
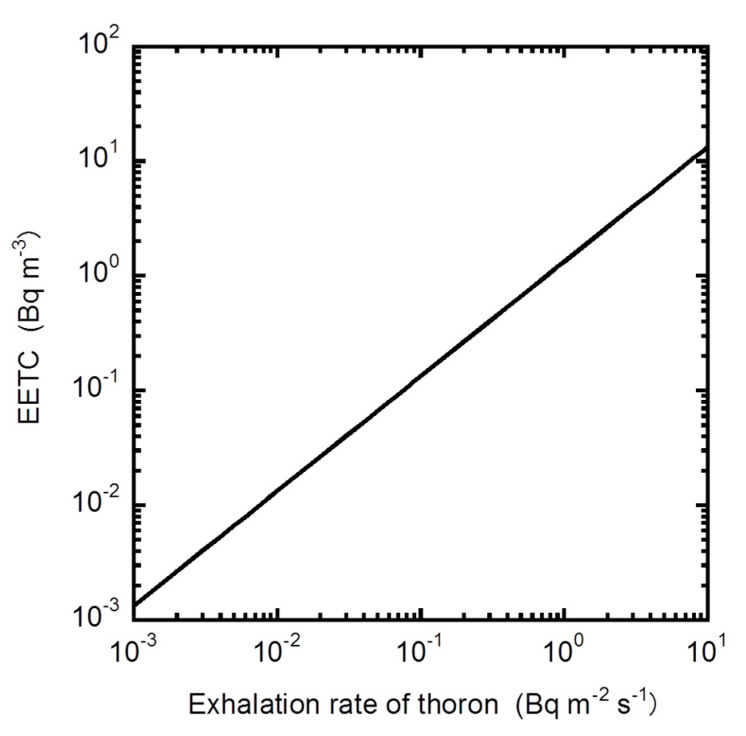
The relationship between Equilibrium Equivalent Thoron Concentration (EETC) and exhalation rate of thoron.

Figure 3 illustrates the behavior of radon/thoron and their progeny in indoor air. After radon and thoron decay, their progenies are formed. Most of these are positively charged and they rapidly capture water molecules, thus forming clusters. They move so quickly in air that some of them attach to ambient aerosols and the others deposit on the wall, ceiling, floor and macro-surfaces. Therefore radon/thoron progeny are generally classified into two fractions: unattached and attached fractions. As unattached progenies have a high diffusive velocity, they deposit on available surfaces very quickly. Even progeny attached to ambient aerosols may eventually deposit on the surface. Before Po-216 atoms are captured by ambient aerosols, they decay to Pb-212 atoms. After considering the half-life of Pb-212, the negligible outdoor Pb-212 activity concentration, and the attachment process to aerosols, Pb-212 activity concentration (*C*_B_ [Bq m^−3^]) in a room can be obtained by the Equation (4):

(4) $$C_{B}=\frac{\lambda_{a}\lambda_{B}E_{\mathrm{Tn}}}{\lambda_{\mathrm{Tn}}\left(\lambda_{B}+\lambda_{v}+\lambda_{d}^{a}\right)\left\{\lambda_{B}+\lambda_{a}+\sqrt{\lambda_{\mathrm{Tn}}\left(\lambda_{B}+\lambda_{a}\right)}\right\}}\cdot \frac{S}{V}$$

where *λ*_B_ is decay constant of Pb-212 [s^−1^]; *λ*_a_ is attachment rate of unattached thoron progeny onto ambient aerosols [s^−1^]; *λ_v_* is ventilation rate of the room [s^−1^]; $\lambda_{d}^{a}$ is deposition rate of attached thoron progeny [s^−1^]; *S* is surface area where thoron atoms are emitted [m^2^]; and *V* is inner volume of the room [m^3^]. Based on the same manner, Bi-212 activity concentration (*C*_C_) is subsequently given by the Equation (5):

(5) $$C_{C}=\frac{\lambda_{C}C_{B}}{\lambda_{C}+\lambda_{v}+\lambda_{d}^{a}}$$

where *λ*_C_ is decay constant of Bi-212 [s^−1^]. When the typical parameters are given in Table 1 [10,12,13], EETC can be estimated with the exhalation rate of thoron as shown in Figure 4. De With et al. [14] reported the thoron exhalation rate from the wall against the EETC value in the room. As the physical parameters except Surface-to-Volume (S-V) ratio are not expected to be much different in any indoor environment, EETC can be simply expressed along with the exhalation rate of thoron and S-V ratio as the Equation (6):

(6) $$\mathrm{EETC}=3.36E_{\mathrm{Tn}}\frac{S}{V}$$

Note that the EETC may change if another value of each parameter is adopted from the range.

## 3. Measurement Techniques

### 3.1. Spot Measurement

#### 3.1.1. Thoron

As the half-life of thoron is shorter than 1 min, thoron gas measurement needs to start immediately after sampling. In this section, the measurement method using one scintillation cell is briefly introduced. Tokonami et al. [15] developed a discriminative measurement technique for radon and thoron concentrations with time-sequential counting. Prior to the measurement, alpha counting efficiencies for radon, ^218^Po, ^214^Po, thoron and ^216^Po were estimated by a Monte Carlo Calculation after taking their range into account based on their emitted energies as well as the size of the cell. In their study, Pylon scintillation cells of 300A and 110A were used. Their inner volumes are 270 [mL] and 151 [mL], respectively. As this technique can be completed within 15 min, contribution from any other alpha emitters of the remaining thoron progeny, such as ^212^Bi and ^212^Po, can be ignored for the determination of thoron concentration. In order to validate justification of the alpha counting efficiencies by the Monte Carlo simulation, the conversion factor theoretically drawn was compared with that experimentally given by the manufacturer. The large cell conversion factor (300A) provided by the manufacturer is the value at radioactive equilibrium, which is given as 27.9 [Bq m^−3^ cpm^−1^]. After radon gas is drawn into the cell, it takes 3.5 h to reach the equilibrium between radon and its progeny. With the latest nuclear data, the theoretical conversion factor is eventually estimated to be 28.3 [Bq m^−3^ cpm^−1^], where there is only a small difference between the two approaches. Zhang et al. made a similar approach in the conversion factor of the same scintillation cell [16]. The alpha counting efficiencies for thoron and its progeny are close to those given by Tokonami et al. [17]. As the theoretical approach has been justified, it can be also applicable to the determination of thoron concentration with the alpha counting efficiencies of thoron and ^216^Po. Furthermore, it can be regarded that ^216^Po atoms behave like a gas in the cell and that thoron and ^216^Po are at equilibrium because its half-life is very short. The thoron concentration (*C*_Tn_ [Bq m^−3^]) is given by the Equation (7):

(7) $$C_{\mathrm{Tn}}=\frac{N_{\mathrm{Tn}}}{V_{c}\times \left(\eta_{\mathrm{Tn}}+\eta_{\mathrm{ThA}}\right)\int_{t_{0}}^{t_{0}+t_{m}}e^{-\lambda_{\mathrm{Tn}}t}dt}$$

where *N*_Tn_ is counts during the period; *V*_c_ is inner volume of the cell [m^−3^]; *η*_Tn_ is counting efficiency of thoron; *η*_ThA_ is counting efficiency of ^216^Po; *t*_0_ is beginning of the measurement [s]; and *t*_m_ is measurement period [s]. If radon is present together with thoron, however, counts derived from radon and its progeny need to be subtracted from *N*_Tn_. In order to obtain net counts derived from thoron and its progeny, another measurement is therefore necessary after thoron and ^216^Po completely decay. *N*_Tn_ can be expressed as the Equation (8):

(8) $$N_{\mathrm{Tn}}=N_{1}-kN_{2}$$

where *N*_1_ is counts during the first period; *N*_2_ is counts during the second period. The constant *k* depends on the existing ratio of radon and its progeny in the cell and the measurement timetable. In the previous study, an optimal timetable with a 15 min time interval was discussed. The following timetable was proposed: twenty seconds after sampling, the first measurement is made over 100 s. Ten minutes after sampling, a 5 min counting, as the second measurement, is made.

#### 3.1.2. Thoron Progeny

The measurement technique for thoron progeny is similar to that for radon progeny. In general, an alpha counting method is preferable. As ^212^Pb and ^212^Bi concentrations are assigned to the subject of dose assessment in thoron progeny measurement, the counting method is simpler than that for radon progeny. Two time-sequential counts are necessary to measure two kinds of thoron progeny concentration in both gross alpha counting and alpha spectroscopic methods. As the half-life of ^212^Pb is as long as 10 h, however, it takes a significant amount of time to measure thoron progeny concentrations precisely. In the gross counting method, a ZnS(Ag) (siliver-activated zinc sulfide) scintillation counting system is commonly used. As this technique has no alpha energy discrimination, however, it will be impossible to complete the determination of thoron progeny concentration in a natural environment because radon will also be present together with thoron. Therefore, the measurement timetable needs to be optimized so as to determine thoron progeny concentrations. Unless radon progeny concentrations are the subject of measurement, the measurement can begin after radon progeny completely decay (practically after 6 h). Note that accuracy of ^212^Bi activity concentration will be diminished when considering the half-life of ^212^Bi (60 min). In order to overcome such practical problems, the least-square method will be suitable. This can give any activity concentrations regardless of the number of unknown concentrations. In contrast, the alpha spectroscopic method can quickly terminate the measurement for both radon and thoron progeny, because the alpha particles emitted from them can be identified due to the high resolution of the alpha spectrum. Information on the highest alpha particle energy emitted from ^212^Po is available via this technique without any interference from any other alpha emitters. When the dose assessment is referred, determination of ^212^Pb will be emphasized because the contribution from ^212^Bi is much smaller than that from ^212^Pb as shown in the Equation (1). Tokonami et al. [17] developed a simple measurement technique for the equilibrium equivalent thoron concentration with a solid-state nuclear track detector. A poly allyl diglycol carbonate (PADC), commercially named CR-39, is used as the detecting material [17]. This passive technique is applicable to determine the radioactivity level anywhere without electricity supply. The following procedure, before chemical etching and track reading, can be introduced for the determination of thoron progeny:

1. Air samples are taken over several hours with a membrane filter (Millipore AA) or glass microfiber filter (Whatman GF/F) installed in an open-faced filter holder and a DC powered air pump;
2. The filter is left until radon progeny completely decay (more than 6 h);
3. An aluminum foil (4.0 mg cm^−2^) as the energy absorber is directly placed on the filter so as to detect alpha energy emitted from thoron progeny, and then a CR-39 plate is attached for alpha track registration;
4. The time is recorded when the CR-39 plate is removed. This is the end of the measurement process.

### 3.2. Continuous Measurement

#### 3.2.1. Thoron

There are two main ways to continuously identify thoron even though radon is present as well. Falk et al. [18] developed a delayed coincidence method. The method separates the fraction of alpha counts emitted from ^216^Po from all the other alpha counts. This method is based on the short half-life of 150 ms of ^216^Po. Bigu and Elliot [19] developed a continuous monitor based on their concept. Although similar monitors were also developed, a flow-through scintillation cell is used in any measurement system. Alternatively, alpha spectrometry is used. A RAD7 monitor, commercially available, is based on an electrostatic collection method (for instance, Takeuchi et al., 1999) [20]. In this monitor, air is drawn into the decay chamber through the drying column. As radon and thoron progeny are positively charged, they will be neutralized by vapor and subsequently will not be collected on the surface of the silicon semiconductor detector as the electrode unless air is dried. In addition, the half-life of ^216^Po is so short that a large mobility will be required by high voltage to obtain a sufficient sensitivity to thoron. The voltage cannot be changed in the above monitor. Therefore, a sampling flow rate is one of the important parameters for thoron sensitivity due to its short half-life. Special attention must be paid to the flow rate when determining thoron concentrations with this monitor.

#### 3.2.2. Thoron Progeny

There are several commercial products for continuous working level monitoring. Note that any signals derived from thoron progeny cannot be separated from those of radon progeny unless alpha spectroscopy is used. In principle, the alpha spectroscopic method can specify information regarding thoron progeny though it cannot determine the concentration. In a specific continuous monitor, the EETC can be simply determined using the count rate (*CPM*) and an experimentally obtained conversion factor (*CF*) as in the Equation (9):

(9) $$\mathrm{EETC}=\frac{CPM}{CF}$$

As the conversion factor is obtained under the condition where the EETC is constant, however, the EETC does not always correspond to an actual variation. On the contrary, a special algorithm for potential alpha energy concentrations (PAEC) developed by Tokonami et al. [21] would be applicable in this case.

### 3.3. Time-Integrated Measurement

#### 3.3.1. Thoron

**Figure 5:**
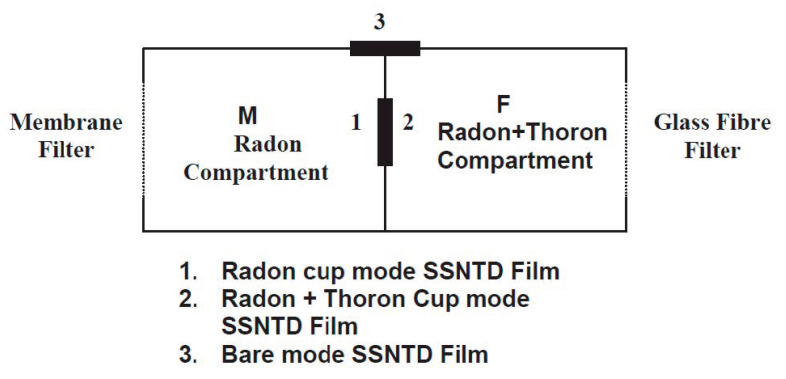
A twin cup radon-thoron discriminative monitor [22].

**Figure 6:**
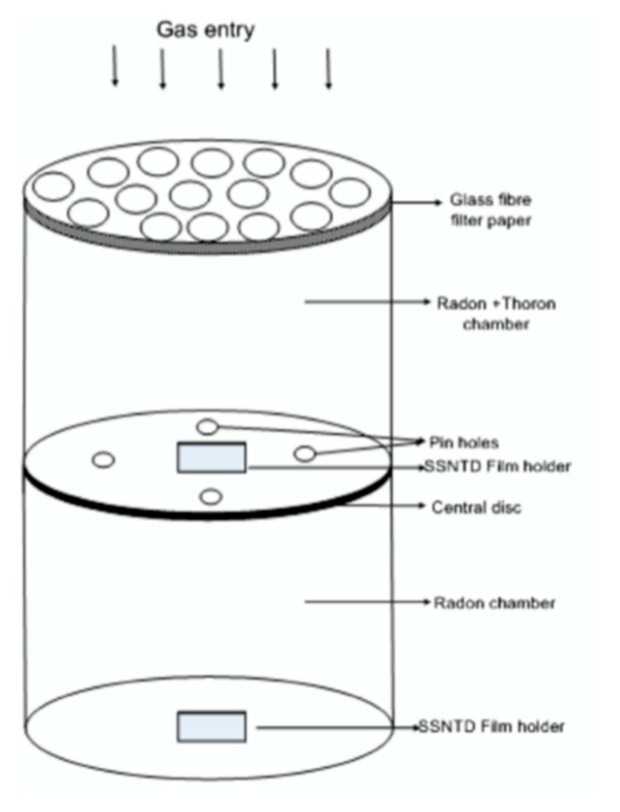
A pin-hole based radon-thoron measurement device [23].

Passive monitors are available for long-term measurement for both radon and thoron. This technique is commonly used in nation-wide or regional surveys. Solid state nuclear track detectors and electrets are installed in such a passive system. As they cannot separate radon and thoron signals, however, a dual measurement system needs to be chosen. This dual system is derived from the large difference of the half-life between two radioisotopes. For this purpose, the system accommodates two different diffusion chambers where detectors are installed and in each the entry rate of gas is well controlled by a gap or filter. In this section, two types of monitor are introduced. Eappen and Mayya developed a twin cup radon-thoron dosimeter [22] (Figure 5). Three pieces of LR-115 Type II detector are fixed in the twin chamber radon dosimeter having three different mode holders. The exposure of the detector is termed as the cup mode whereas the one exposed as open is termed the bare mode. The right chamber is covered with a glass fiber filter and therefore both radon and thoron gases can easily enter the chamber. The left chamber is covered with a membrane filter so as to reduce the entry of thoron. Thus, there is less sensitivity for thoron in the left chamber than the right chamber. The third detector film exposed in the bare mode registers alpha tracks contributed by concentrations of radon, thoron and their progeny. Thereafter another type of passive monitor (Figure 6) was developed by Sahoo et al. [23]. A pin-hole based ^222^Rn/^220^Rn discriminator was installed in the monitor. For discriminative measurement of two radon isotopes, a pin-hole diffusion barrier was used [24,25]. This is because different entry rates of ^222^Rn were pointed out through two entrances of the dosimeter which might arise from turbulence or air flow in one direction. The new device was designed to overcome the limitation of the conventional twin cup dosimeter. Currently this pin-hope monitor has been widely used in India.

**Figure 7:**
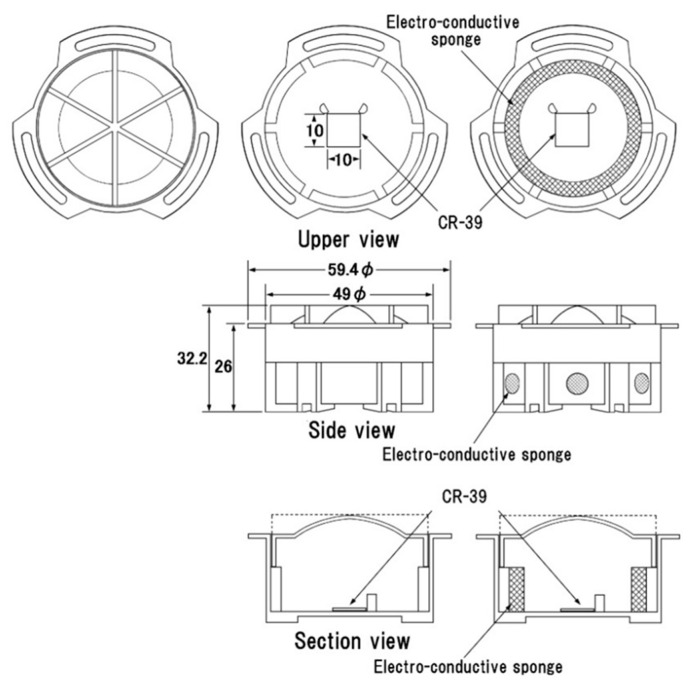
A passive type radon (^222^Rn) and thoron (^220^Rn) discriminative monitor [26].

Tokonami et al. [26] also developed a passive ^222^Rn and ^220^Rn discriminative monitor for a large-scale survey (Figure 7). The measurement principle is almost the same as the Indian monitor except for their bare mode. PADC, commercially CR-39, is used as the detecting material. This monitor and its prototype have been widely used in various countries [27,28,29,30]. The above two monitors can be calibrated in the calibration chamber at Hirosaki University Institute of Radiation Emergency Medicine, Japan [31]. For determination of radon and thoron activity concentrations with passive solid-state nuclear track detectors, ISO 16,641 [32] is currently available. The detection threshold, detection limit and confidence lower/upper limits in this technique are calculated based on ISO 11,929 [33]. A comparative performance test of Indian and Japanese-Hungarian monitors was carried out in the environment [34].

#### 3.3.2. Thoron Progeny

**Figure 8:**
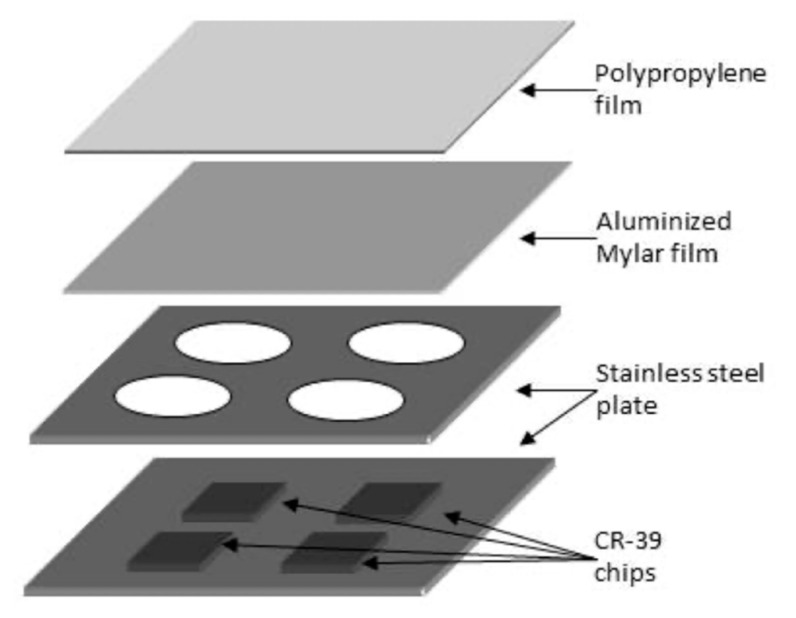
An overview of a thoron progeny monitor [11].

**Figure 9:**
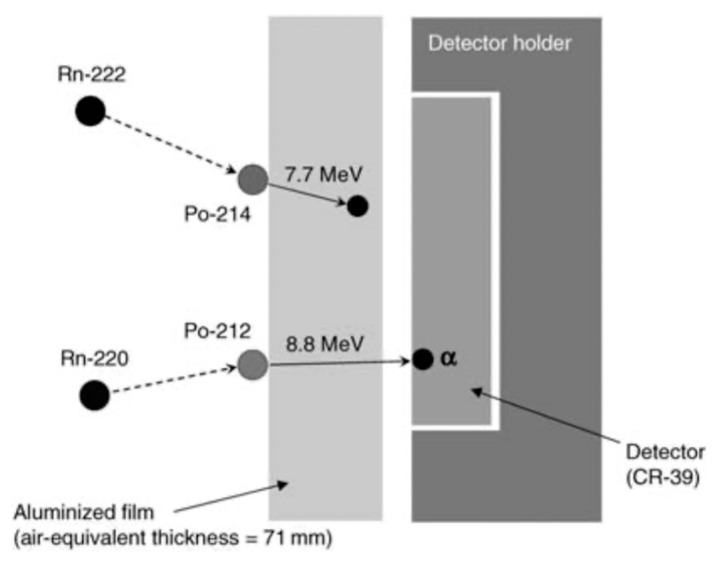
A detecting principle of alpha energy emitted from ^212^Po [36].

A prototype of the passive type thoron progeny monitor was developed by Zhuo and Iida based on diffusive deposition on the surface [35]. Among thoron progeny, ^212^Po atoms emit alpha energy of 8.8 MeV, which is the highest alpha energy of all the natural radionuclides. It is, hence, obvious that it will be easy to detect this high energy by separating different energies emitted from other radionuclides if an alpha energy absorber with a proper thickness is prepared. Figure 8 shows an overview of a thoron progeny monitor. For radiation detection, CR-39, one of the solid-state nuclear track detectors, is mounted in the monitor. The body is made of stainless steel. As shown in Figure 8, four pieces are installed in the monitor and they are covered with an aluminized Mylar film and a polypropylene film in this order (thickness: 7.1 mg cm^−2^; air-equivalent thickness: 71 mm). By adjusting the thickness properly, only alpha energy of 8.8 MeV can be detected. Figure 9 exemplifies the detecting principle of alpha energy emitted from ^212^Po [36].

The monitor is hung on the wall for a certain period. In a usual survey, it is exposed for a few months. Radon and thoron progeny in indoor air deposit on the wall over the time period. After they are deposited, tracks of alpha particles are recorded in the CR-39. After retrieving the monitors, they are chemically etched to identify alpha tracks with a track reading system. The etching condition for CR-39 (Baryotrak; Nagase Landauer Ltd., Japan) is as follows: solution: 6.0 M NaOH; temperature: 60 °C; time: 24 h. Using a track reading system such as a microscope, track density is determined. The relationship between track density (*D*) and thoron progeny concentration, i.e., equilibrium equivalent thoron concentration, is expressed as the Equation (10):

(10) $$\mathrm{EETC}=\frac{D}{C\times T}$$

where *D* is track density (tracks mm^−2^); *C* is conversion factor experimentally obtained (0.017 tracks mm^−2^ (Bq m^−3^ day)^−1^ in our monitor); *T* is exposure period (day); and EETC: equilibrium equivalent thoron concentration (Bq m^−3^).

The conversion factor was experimentally obtained by the comparison between the monitor and intermittent thoron progeny measurement. The experiment was carried out in actual dwellings. Using the proposed technique, the lowest detection limit of the EETC is estimated to be 0.005 Bq m^−3^ with 90-day exposure.

Similar techniques were found in Indian studies [37,38,39,40,41,42,43]. Instead of CR-39, LR-115 nuclear track detectors are used in their monitors. Not only thoron progeny sensors but also radon progeny sensors are installed by differentiating the thickness of energy absorbers. Furthermore, metal wire screens are introduced to detect fine and coarse progeny aerosols separately [44].

## 4. Dosimetry

When assessing the annual effective dose due to radon/thoron progeny inhalation, dose conversion factors are used. International bodies such as UNSCEAR and ICRP have their own values. The dose conversion factors (DCF) for radon are derived from both epidemiological evidence and dosimetric models, whereas the DCF for thoron is given only by the dosimetric model because there is no epidemiological evidence of lung cancer incidence due to thoron progeny inhalation. Table 2 summarizes effective dose conversion factors (mSv WLM^−1^) (WLM: Working Level Month) for thoron. The DCF for thoron is about two–three times smaller than that for radon [1,45,46,47,48,49,50,51]. When rewriting the DCF, expressed in dose per unit equilibrium equivalent activity concentration of thoron/radon exposures, however, the DCF for thoron is more than two times larger than that for radon. According to the latest DCF for thoron and radon in ICRP Publ. 137 [51], they can be given as 107 nSv (Bq h m^−3^)^−1^ and 16.8 nSv (Bq h m^−3^)^−1^, respectively. On the contrary, UNSCEAR has recently decided to use the conventional values of 40 nSv (Bq h m^−3^)^−1^ and 9 nSv (Bq h m^−3^)^−1^, respectively, despite the inconsistency. This needs more consideration in order for them to correspond each other.

## 5. Radioactivity and Resulting Dose

As mentioned above, thoron activity concentration is not uniformly distributed in the environment, which is far from the case for radon. It is considered that thoron concentration in air exponentially decreases with distance from the source. This behavior is derived from the very short half-life of thoron (55.6 s). The exponential change of thoron concentration is defined via the diffusion coefficient, strongly affected by the air turbulence condition.

Table 3 summarizes thoron and thoron progeny concentrations (EETC) in various countries. As can be seen from the presented data, their number is more restricted than that of radon [27,39,41,42,52,53,54,55,56,57,58,59,60,61,62,63,64,65,66,67].

In Cameroon, radon, thoron and its progeny concentrations were measured in residential areas in uranium and thorium bearing regions [52]. UNSCEAR presents the typical value of the equilibrium factor of thoron as 0.02 and this equilibrium factor of thoron is often used to estimate the annual effective dose due to thoron, in the same manner as in the case of radon. In the present study, the authors estimated a total annual effective dose derived from radon and thoron using actual measurement data on thoron progeny and compared it with that given by the UNSCEAR method. Consequently, the result based on the direct measurement was 1.5 times larger than the indirect one. They concluded that the direct measurement of thoron progeny is important for dose assessment.

The results of two surveys in Canada were tabulated. In one survey, long-term thoron and progeny measurements were simultaneously carried out for three months in two cities [53]. The simultaneous measurement of thoron and thoron progeny concentrations yielded a thoron equilibrium factor of 0.002 and therefore the authors concluded that the typical value given by UNSCEAR is reasonable for dose assessment. In contrast to a Cameroonian study, the Canadian study justified the consistency of the thoron equilibrium factor via the UNSCEAR method. In the other survey, results of simultaneous radon and thoron measurements were shown in 33 metropolitan areas [54]. The study demonstrates that thoron contributes around 3% of the effective dose due to indoor radon and thoron exposure in Canada.

The US National Cancer Institute and the China Ministry of Health conducted an epidemiological survey for residential radon and lung cancer in Gansu Province, China [68]. This study can be recognized as one of the main studies of residential radon by pooling the analyses of European [69,70], North American [71,72] and Chinese [73] residential case-control studies. They used alpha track detectors, but the monitors were proved to be influenced by thoron and overestimated radon concentrations [74]. Thereafter radon measurements were made with the above mentioned improved detectors, discriminating two radon isotopes [56]. Remarkably high thoron levels were observed in these areas. This finding suggests two key points, as follows: (1) their previous radon data and the lung cancer risk were incorrect; (2) the thoron contribution to radiation exposure will be important in those areas. Another Gansu survey was conducted with simultaneous measurements of radon, thoron and thoron progeny [75]. Correlation analyses were made among three activity concentrations. There was no correlation whenever any two concentrations were chosen. This means that these three concentrations are so independent that it is difficult to estimate one concentration from the other. If the thoron dose needs to be considered, direct measurement of thoron progeny is required. This further implies that thoron progeny concentration cannot be accurately obtained with a fixed thoron equilibrium factor.

Simultaneous measurements of radon, thoron and thoron progeny were made in other provinces close to Gansu province, namely Shanxi and Shaanxi provinces [57]. From the topographical and geological points of view, the same radiological features were obtained. Compared to thoron concentrations, thoron progeny concentrations were so low that it resulted in small thoron equilibrium factors (arithmetic mean = 0.01). Tokonami [36] evaluated the influence on the risk estimate of misleading radon data. Annual effective doses due to radon and thoron were estimated in the UNSCEAR manner. Comparison of the annual effective dose was made between misleading radon concentrations and modified, i.e., to achieve correct radon concentrations. Misleading radon concentrations resulted in an arithmetic mean of 6.4 mSv, whereas correct ones gave 1.7 mSv. When the contribution of thoron was included, the total dose was calculated to be 2.4 mSv. A series of these findings revealed that the Gansu study gave incorrect or misleading lung cancer risk estimates.

Yangjiang, Guangdong province, is famous for being one of the areas with the highest background radiation in the world. Kudo et al. [55] demonstrated how residents there are being exposed to natural radiation. As monazite sands are widely distributed in this area, high gamma dose rates are often observed. However, there is less information on internal exposure, particularly due to radon and thoron. Based on collected data on these activity concentrations using the UNSCEAR method, annual effective doses due to radon and thoron progenies were estimated to be 3.1 (SD = 2.0) mSv and 2.2 (SD = 2.5) mSv, respectively. This revealed that indoor thoron and its progeny levels were fairly high and even thoron exposures are not negligible compared to radon exposures.

Kovacs [58] summarized radon and thoron surveys in Hungary. Dwellings and workplaces were surveyed with passive radon-thoron discriminative monitors. The monitors were placed 15–30 cm from the wall. Table 2 gives examples of thoron concentrations observed in underground bauxite mines. It was concluded that the dose contribution from thoron progeny was not negligible considering all the data and consequently further surveys of thoron progeny would be required for accurate dose assessment.

Omori et al. [59] presented radon, thoron and progeny concentrations for dwellings in Kerala, India. Their study area was classified into high (3–5 mGy y^−1^) and low (1 mGy y^−1^) background radiation areas, respectively. In a six-month measurement, it was found that there was no major difference between the two areas. The geometric mean of the annual effective dose due to radon and thoron was estimated to be 0.10 and 0.44 mSv, respectively. The internal dose derived from thoron progeny is more significant than that from radon. However, the doses were quite small and the external dose can be regarded as the major contributor in Kerala.

Omori et al. [27] also conducted long-term measurements of indoor radon, thoron and thoron progeny concentrations in Odisha, India. They revealed that radon and thoron concentrations differ by one order of magnitude whereas thoron progeny concentrations were nearly constant throughout the whole year. Thoron and its progeny concentrations were higher than those in Kerala. Exposure to thoron is equal to or exceeds exposure to radon in internal doses. The internal dose from radon and thoron was comparable to the external dose.

In Ireland, indoor concentrations of radon, thoron and its progeny were measured in 205 dwellings during the period 2007–2009 [60]. Radon activity concentration ranged from 4 to 767 Bq m^−3^ with an arithmetic mean of 75 Bq m^−3^. Based on these concentrations and the UNSCEAR approach, the corresponding estimated annual effective doses are 0.1 (min), 19.2 (max) and 1.9 (mean) mSv. On the other hand, the estimated annual effective doses corresponding to thoron progeny concentrations are 2.9 (max) and 0.35 (mean) mSv with the dose conversion factor based on the two dosimetric models [39,40]. Although the dose from thoron tends to be negligible in most cases worldwide, it should be noted that in some dwellings in this study the annual dose from thoron progeny exceeded that from radon. This result is the first case where two annual effective doses from radon and thoron were measured on a nationwide scale in Europe.

Nyambura et al. [61] carried out indoor radon, thoron and thoron progeny surveys in several different types of houses in Kilimambogo, Kenya, and thereafter assessed the annual effective dose attributed to inhalation of their progeny. Housing structure was classified into three categories, i.e., mud, metal and stone-walled houses. The highest mean thoron and its progeny concentrations were observed in mud-walled houses with 195 and 11.5 Bq m^−3^, respectively, whereas the highest radon concentration was found in stone-walled ones with 75 Bq m^−3^. Assessing the annual effective dose, the highest was given by mud-walled houses with 0.9 (min), 8.5 (max) and 3.7 (mean) mSv, respectively.

Activity concentrations of thoron and its progeny were measured in 450 houses from 2002 to 2004 in Korea [62]. The annual arithmetic and geometric means of thoron concentration were 40.4 and 10.7 Bq m^−3^, respectively. The annual arithmetic and geometric mean were 0.89 and 0.60 Bq m^−3^, respectively. High thoron concentrations were observed in Korean-style houses built with mud block. The average annual effective dose due to inhalation exposure to thoron and its progeny was estimated to be 0.25 mSv.

Indoor thoron concentrations were measured in 300 houses for one year, from December 2008 to December 2009 in Macedonia. using passive radon-thoron discriminative monitors [63]. They were deployed at a distance of more than 50 cm from walls. The geometric means of indoor thoron concentration in winter, spring, summer and autumn were obtained as 39 (3.4), 32 (2.8), 18 (2.8) and 31 Bq m^−3^ (2.9), respectively. Seasonal variations of thoron appear to be smaller than those of radon.

Indoor thoron concentrations in 50 houses were measured in the Metropolitan Zone of Mexico City using a passive electret system [64]. The annual arithmetic and geometric means of indoor thoron concentration were estimated to be 82 and 55 Bq m^−3^, respectively, ranging from 8 to 234 Bq m^−3^. As to the seasonal variation, the lowest value was found in summer.

Thoron progeny concentrations, namely equilibrium equivalent thoron concentrations (EETCs), were measured in 2900 houses, Netherlands [65]. The arithmetic mean of EETC was 0.64 Bq m^−3^. Thoron progeny concentrations show correlations with year of construction and smoking behavior. A pilot study was also conducted to determine the relationship between the exhalation of thoron and the concentration of thoron progeny in the room. The authors pointed out that thoron might be a more important contributor to the population dose in other regions with low radon levels.

A limited number of measurements were carried out about 1 m away from any wall and 1.5 m above the floor in various environments in Slovenia using passive radon-thoron discriminative monitors [66]. Thoron and radon concentrations in 35 elementary schools ranged from 21 to 368 and 40 to 4609 Bq m^−3^, respectively. The authors pointed out that there was a weak correlation between the two activity concentrations though both of them followed a lognormal distribution.

Results of the first investigation on indoor radon, thoron and their progeny concentrations were given in 25 primary schools of Republic Srpska [41]. For their measurements, Japanese and Indian techniques were introduced in the survey. The monitors were deployed at 10 cm distance from the wall. A weak correlation was found between radon and thoron concentrations as well as thoron and thoron progeny concentrations.

Gulan et al. [39] carried out indoor radon, thoron and their progeny survey in scattered locations around Kosovo. Estimated arithmetic mean values of concentrations in 48 houses are 122 Bq m^−3^ for radon and 136 Bq m^−3^ for thoron. This might be attributed to building materials involving bricks, sand and stones from the local area where ^232^Th concentration in soil is higher than that of ^226^Ra.

Simultaneous long-term measurements of radon, thoron and their progeny were conducted in 40 rural houses in Serbia [42]. The EETC was found to be relatively higher than the worldwide average value. Significant positive correlation between thoron and EETC was found, whereas there was no significant correlation between radon and EERC.

Recently, a high natural background radiation area (HNBR) due to terrestrial radiation has been reported in West Sulawesi, Indonesia [76]. EETC was measured using the thoron progeny monitor shown in Figure 4 in a total of 45 dwellings [67]. The EETC ranged from 4 to 40 Bq m^−3^ and the annual effective dose due to thoron inhalation was reported to be 5.1–17.7 mSv.

Future authors should discuss these results and how they can be interpreted from the perspective of previous studies and working hypotheses. The findings and their implications should be discussed in the broadest context possible. Future research directions may also be highlighted.

## 6. Conclusions

As thoron is a very short half-lived radionuclide, though it is an isotope of radon, it is not easy to measure its activity in air and consequently to assess the resulting dose in the same manner as for radon. Nationwide indoor radon surveys have been conducted in many countries. The annual effective dose for the public is calculated using the indoor radon concentration and an equilibrium factor for radon. The equilibrium factor of radon is typically 0.4 but such an approach is not applicable or meaningful in the case of thoron. The spatial distribution of thoron is so unique that a single value of thoron concentration cannot be given even in a room, due to the short half-life of less than 1 min. Thus, thoron concentrations should not be used for radiation protection purposes because the thoron concentration varies widely with space. Therefore, a direct measurement of thoron progeny concentration will be more effective and useful whereas several assumptions are required in the measurement techniques presented in this paper. As another approach, the surface exhalation rate of thoron may be an index for thoron dose assessment. Although thoron was underestimated in the past, recent findings have revealed that reassessment of risks due to radon exposure may need to take the presence of thoron into account.

## Figures and Tables

**Table 1:** Physical parameters for indoor model [10,12,13].

Parameter ^1^	Range	Typical
Decay constant of thoron [h^−1^]	-	44.74
Decay constant of Pb-212 [h^−1^]	-	0.065
Attachment rate of unattached thoron progeny onto ambient aerosols [h^−1^]	3–110	50
Ventilation rate of the room [h^−1^]	0.1–1	0.5
Deposition rate of attached thoron progeny [h^−1^]	0.015–0.35	0.2
Surface-to-Volume ratio [m^−1^]	-	0.36

^1^ Unit is expressed in h^−1^ so as to easily compare with previous studies.

**Table 2:** Summary of effective dose conversion factors for thoron.

References	Effective Dose Conversion Factors (mSv WLM^−1^) ^1^
Marsh and Birchall [45,46]	3.8
UNSCEAR [1]	1.9
Porstendoerfer [47]	2.4
Ishikawa et al. [48]	5.4
Kendall and Phipps [49]	5.7
Hofmann et al. [50]	4.6
International Commission on Radiological Protection (ICRP) Publ. 137 [51]	5.6 (Indoor workplace) 4.8 (Mine)

^1^ Working Level Month (WLM) is a historical unit of alpha potential energy exposure. 1 WLM = 3.45 mJ h m^−3^.

**Table 3:** Thoron and thoron progeny concentration (EETC) in various countries.

Country		Thoron (Bq m^−3^)	EETC (Bq m^−3^)	Remarks	Reference
Cameroon	AM ^1^	173 (13)	10.7 (0.9)		[52]
	GM ^2^	118 (6)	7.4 (4.8)		
	Range	23–724	0.4–37.6		
Canada	AM ^1^	114 (303)	1.23 (1.51)	Halifax and Fredericton	[53]
	GM ^2^	51 (2.93)	0.75 (2.64)		
	Range	6–1977	0.11–7.45		
Canada (33 metropolitans)	AM ^1^	9 (11)	-		[54]
	Range	ND–164	-		
China (Yangjiang)	AM ^1^	1247 (1189)	7.8 (9.1)		[55]
	Median	859	4.2		
	Range	65–3957	0.6–36.2		
China (Gansu)	AM ^1^	433 (210)	-		[56]
	GM ^2^	347 (2.29)	-		
	Range	19–820	-		
China (Shanxi)	AM ^1^	160	1.4		[57]
	GM ^2^	130 (2.0)	1.2 (1.8)		
China (Shaanxi)	AM ^1^	202	2.3		[57]
	GM ^2^	181 (1.6)	2.1 (1.6)		
Hungary	GM ^2^	341 (2.59)	-	Bauxite mine	[58]
	Range	40–2514	-		
India (Kerala)	GM ^2^	41	1.81 (1.9)		[59]
	Range	11–212	0.36–8.00		
India (Odisha)	AM ^1^	123 (105)	3.19 (2.75)		[27]
	GM ^2^	95 (1.95)	2.37 (2.15)		
	Range	15–585	0.44–15.40		
Ireland	AM ^1^	22	0.47		[60]
	Range	<1–174	<0.05–3.8		
Kenya	AM ^1^	195 (36)	11.5 (2.1)		[61]
	Range	BDL–973	0.8–29.1		
Korea	AM ^1^	40 (56)	0.89 (0.70)		[62]
	GM ^2^	11 (2.9)	0.6 (0.41–0.78)		
	Max	731	-		
Macedonia	AM ^1^	37 (36)	-		[63]
	GM ^2^	28 (2.12)	-		
	Range	3–272	-		
Mexico	AM ^1^	82 (75)			[64]
	GM ^2^	55			
	Range	8–234			
Netherlands	AM ^1^	-	0.64		[65]
	95-Percentile	-	1.37		
	Max	-	13.3		
Slovenia	AM ^1^	87	-	Elementary School	[66]
	Range	21–368	-		
Srpska	AM ^1^	63 (40)	0.52–0.34		[41]
	GM ^2^	51 (2.07)	0.40 (2.20)		
	Range	7–198	0.09–1.16		
Kosovo	AM ^1^	136	2.06		[39]
	GM ^2^ Range	90 18–1313	1.90 0.87–4.38		
Serbia	AM ^1^	116	1.1		[42]
	GM ^2^	89	0.86		
	Range	10–412	0.1–3.4		
Indonesia	AM ^1^	152 (indoor)	13 (indoor)	West Sulawesi (HNBR)	[67]
		139 (outdoor)	15 (outdoor)		
	GM ^2^	141 (indoor)	13 (indoor)	Number of dwellings	
		121 (outdoor)	15 (outdoor)		
	Range	20–618 (indoor)	4–40 (indoor)	Indoor: 45	
		23–457 (outdoor)	4–37 (outdoor)	Outdoor: 18	

^1^ AM: Arithmetic mean, ^2^ GM: Geometric mean.
